# Micro-RNA Implications in Type-1 Diabetes Mellitus: A Review of Literature

**DOI:** 10.3390/ijms222212165

**Published:** 2021-11-10

**Authors:** Kosmas Margaritis, Georgia Margioula-Siarkou, Styliani Giza, Eleni P. Kotanidou, Vasiliki Regina Tsinopoulou, Athanasios Christoforidis, Assimina Galli-Tsinopoulou

**Affiliations:** 1 Unit of Paediatric Endocrinology and Metabolism, Unit of Diabetes Mellitus of Children and Adolescents, 2nd Department of Paediatrics, School of Medicine, Faculty of Health Sciences, Aristotle University of Thessaloniki, AHEPA University Hospital, 54636 Thessaloniki, Greece; kosmas.d.margaritis@gmail.com (K.M.); stellagiza@yahoo.com (S.G.); epkotanidou@gmail.com (E.P.K.); vasotsino@gmail.com (V.R.T.); 2 2nd Department of Obstetrics and Gynaecology, School of Medicine, Faculty of Health Sciences, Aristotle University of Thessaloniki, Ippokratio General Hospital, 54642 Thessaloniki, Greece; gmargioulasiarkou@gmail.com; 3 1st Department of Paediatrics, School of Medicine, Faculty of Health Sciences, Aristotle University of Thessaloniki, Ippokratio General Hospital, 54642 Thessaloniki, Greece; christoforidis@auth.gr

**Keywords:** micro-RNA, type-1 diabetes mellitus, hyperglycemia, biomarkers

## Abstract

Type-1 diabetes mellitus (T1DM) is one of the most well-defined and complex metabolic disorders, characterized by hyperglycemia, with a constantly increasing incidence in children and adolescents. While current knowledge regarding the molecules related to the pathogenesis and diagnosis of T1DM is vast, the discovery of new molecules, such as micro ribonucleic acids (micro-RNAs, miRNAs), as well as their interactions with T1DM, has spurred novel prospects in the diagnosis of the disease. This review aims at summarizing current knowledge regarding miRNAs’ biosynthesis and action pathways and their role as gene expression regulators in T1DM. MiRNAs follow a complex biosynthesis pathway, including cleaving and transport from nucleus to cytoplasm. After assembly of their final form, they inhibit translation or cause messenger RNA (mRNA) degradation, resulting in the obstruction of protein synthesis. Many studies have reported miRNA involvement in T1DM pathogenesis, mainly through interference with pancreatic b-cell function, insulin production and secretion. They are also found to contribute to β-cell destruction, as they aid in the production of autoreactive agents. Due to their elevated accumulation in various biological specimens, as well as their involvement in T1DM pathogenesis, their role as biomarkers in early preclinical T1DM diagnosis is widely hypothesized, with future studies concerning their diagnostic value deemed a necessity.

## 1. Introduction

Type-1 diabetes mellitus (T1DM) is a complex metabolic disease characterized by pancreatic β-cell destruction, which results in insulin secretion deprivation and subsequently hyperglycemia [1]. In 2010, the estimated worldwide prevalence of diabetes among adults was 6.4%, and it is projected to rise to 7.7% by 2030 [2]. T1DM is unquestionably one of the most common chronic diseases of childhood and represents 10% of all cases of diabetes, as well as the most frequent type of childhood and adolescent diabetes, with a rate of up to 90% and a worldwide incidence that is increasing by 3% annually [1,3,4]. Various mechanisms have been incriminated in the pathogenesis of T1DM, including autoimmunity, genetic susceptibility and implication of epigenetics [5]. Autoantibodies targeted against β-cell antigens, specifically against insulin, glutamic acid, decarboxylase, zinc cation efflux transporter and tyrosine phosphatases-2 and -2b, have been detected since T1DM first appeared [5,6,7]. Furthermore, mutations in more than 50 genetic loci have been identified and related to disease susceptibility, with those in human leukocyte antigen (*HLA*) class II gene being the most frequent, followed by variable number tandem repeats (VNTRs) located in the insulin (*INS*) gene, as well as mutations in the cytotoxic T-lymphocyte associated protein 4 (*CTLA-4*) gene [3]. Environmental factors such as viral infections, nutrition and epigenetic factors acting directly in insulin genes and in genes related to autoimmune agents, have also been cited as etiopathogenetic factors affecting disease onset [8,9,10].

Various methods have been widely used in the diagnosis of T1DM, with evaluation of the presence of islet autoantibodies as biomarkers of disease progression being the routine practice. However, most of the diagnostic tools available are capable of confirming the diagnosis of T1DM after the onset of clinical symptoms, thus the emergence of novel biomarkers that function as disease predictors at a preclinical stage is deemed a necessity. Recently, a new group of molecules, micro ribonucleic acids (micro-RNAs, miRNAs), has been identified in various biological samples, and studies report their involvement in T1DM pathogenesis, as well as their potential role in the diagnosis of T1DM via gene expression regulation. This regulation occurs in various biological processes, such as cellular differentiation, proliferation and apoptosis and specifically protein synthesis obstruction, via either translation inhibition or messenger RNA (mRNA) degradation [11,12].

The objective of the present review of literature was to explore the biological pathways involved in miRNA biosynthesis and regulatory function, as well as their potential involvement in T1DM pathogenesis, and therefore enhance current knowledge about their potential diagnostic role in the preclinical stage of the disease.

## 2. MiRNAs

MiRNAs are a group of small noncoding RNAs with a length up to 20 nucleotides, which act as gene expression regulators in a post-transcriptional stage, either directly by modulating gene expression, or indirectly by leading in mRNA deterioration [13].

### 2.1. History

The first formal use of the term miRNA was made by Ambros’s and Ruvkun’s laboratories in 1993, when the lin-4 molecule was documented as the first discovered miRNA molecule. It consisted of a gene located in the nematode Caenorhabditis elegans, and it regulated the temporal development pattern of all larval stages [14,15]. In subsequent years, a second gene region was identified as a micro-RNA gene, by Reinhart et al. The lethal-7 (*let-7*) is also located in the nematode Caenorhabditis elegans and controls the transition from L4 to adult larval development; considering the fact that *let-7* was observed in several species including humans, a wide variety of diagnostic and therapeutic possibilities appeared [16,17,18]. Since then, many studies have been conducted in order to further investigate the presence and role of these molecules in human genes, their regulatory role in expression, and their involvement in molecular pathways and, consequently, disease pathogenesis.

### 2.2. MiRNA Genomic Distribution

Since miRNAs were first discovered, many efforts have been made to further identify miRNA genes in the human genome. Many studies followed and reported that these genes are located in all human chromosomes with the exception of the Y chromosomes. MiRNA genes tend to accumulate in clusters, which later are transcribed as polycistronic primary transcripts. These clusters contain miRNAs related to each other, although it is yet to be determined whether they are also functionally related, regulating similar biological pathways [19,20]. Recent data from studies state that most miRNA genes are located in defined transcription units (TUs), and they can be categorized in three groups base on location: intronic miRNA in protein coding TUs, intronic miRNA in noncoding TUs and exonic miRNA in noncoding TUs [20,21].

### 2.3. Biogenesis Pathway and Maturation of miRNAs

**Figure 1:**
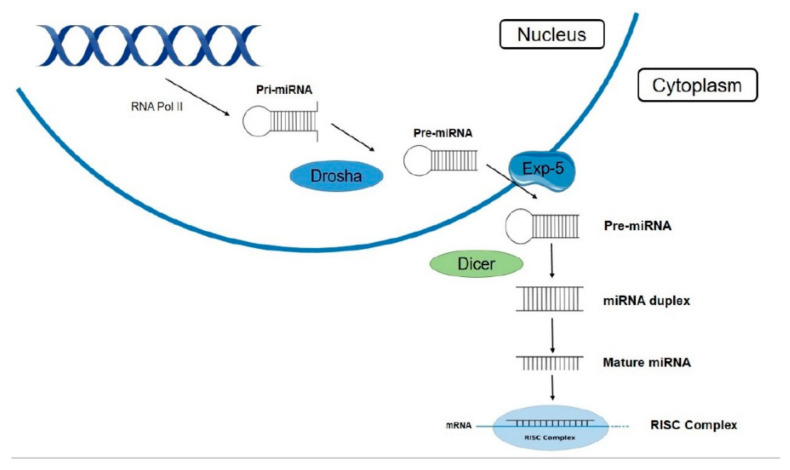
MiRNA biosynthesis and maturing pathway. Biosynthesis of miRNA begins with production of pri-miRNA after the miRNA gene is translated via RNA polymerase II and afterwards pre-miRNA results from splicing of the stem loop miRNA molecule via Drosha protein. Exportin-5 is a protein that allows pre-miRNA to exit the nucleus and to enter the cytoplasm. Dicer protein further splices pre-miRNA into the miRNA duplex molecule, having cut the loop and after a degradation of one strand, the single stranded mature miRNA molecule is produced. It is then incorporated into the RISC complex, inside which the mRNA target is degraded. DNA double strand and protein image from smart.servier.com (accessed on 15 September 2021).

The first step of the biogenesis pathway, leading in the production of mature miRNA molecules, is the transcription of miRNA genes by RNA polymerase II (Pol II). MiRNAs are transcribed as long primary transcripts (pri-miRNAs) containing a stem-loop structure. These transcripts include a cap structure at the 5′ end and a poly (A) tail at the 3′ end, which act as specific targets for Pol II, as well as miRNA core promoters containing elements identified by Pol II, such as the TATA box. Pri-miRNAs undergo cleavage of their stem-loop structure inside the nucleus by an RNase III endonuclease known as Drosha, and the precursor miRNA (pre-miRNA) is then released. This pre-miRNA is a 60–70 nt stem-loop molecule with a 5′ phosphate and a 3′ overhang. The following stages of miRNA biosynthesis are performed outside the nucleus, in the cytoplasm. Pre-miRNA is transported across the nuclear membrane by a specific protein, exportin-5. This transporter is a Ran-dependent nuclear transport receptor that recognizes the loop pattern of pre-miRNA and carries it inside the cytoplasm. Further processing of pre-miRNA by a cytoplasmic RNase, Dicer, produces a double-stranded miRNA, by cleaving the stem loop, and later it is unwound into two single-stranded miRNAs, one strand of which is promoted into the mature miRNA while the other is degraded. The mature single-stranded miRNA is incorporated into large ribonuclear complexes, known as RNA-induced silencing complexes (RISCs) (Figure 1). The double- to a single-strand conversion of miRNA occurs inside these complexes, and their structure allows miRNAs to regulate gene expression by binding into specific miRNA targets [15,20,22,23,24].

### 2.4. Mechanism of Action

Following clarification of the biosynthesis of miRNA molecules, their maturation process and the miRISC complex coming into being, efforts turned to investigating their main mechanisms of action, as well as their effects on gene expression pathways. Their main function was well-established after many studies and consists of the inhibition of protein synthesis, either by obstructing translation of coding genes or by causing mRNA degradation. This main task is achieved by stalling or inhibiting translation, or by mRNA cleavage, prevention of RNP transport to the protein manufacturing unit, or by forging protein complexes alongside ribosomes, enabling them to degrade emerging polypeptides [22]. The majority of these actions are performed through the miRISC complex, a unit that contains an Argonaute protein capable of nucleic slicing and facilitating the interactions between miRNAs and their targets. The outcome of the miRNA-target interaction is determined by the degree of base complementarity. Perfect complementarity of interaction results in mRNA cleavage and deterioration, whereas a lower degree of complementarity between bases leads to translation being repressed. Some data suggest that the dominant mechanism of miRNA action, in regulating gene expression, is that of mRNA destabilization, as most interactions are imperfect and target slicing cannot occur. Further investigation exhibited that miRNAs tend to bind to a specific sequence at the 3′ untranslated region (UTR) end of their targets, causing deadenylation, decapping and consequently translational repression. Various target binding sites have been identified through the years, apart from the 3′ UTR end, with the 5′ UTR end of target mRNAs and coding sequences of genes also being some of them [16,22,23]. The main location where miRNA functions is the cytoplasm. However, it has been reported that specific miRISCs with lower molecular weight have the ability to interact with mRNAs within the nucleus, causing translation depletion. Another possibility is that miRISC complexes are involved in methylation sequences, compactification of chromatin and genomic remodeling [23,25]. Moreover, a supplementary mechanism of miRNA action, apart from pairing with mRNA targets, is that of a decoy molecule, interfering in that manner with regulatory proteins, initiating functional disruption. MiRNAs, as mentioned before, lead to inhibition of gene expression in various ways. Some studies followed a different approach and stated that several miRNAs were found to target gene promoters and activate translation of mRNAs in specific conditions, such as cell cycle arrest and amino acid starvation [23,26,27,28,29].

### 2.5. MiRNAs’ Role as Biomarkers

Many studies investigated the implications of miRNAs in the pathogenesis of several diseases, following the elucidation of their mechanism of action. Due to their key roles in various elementary biological processes such as cellular proliferation, metabolism and death and intracellular communication, their involvement in pathological and possibly malignant outcomes became obvious. MiRNAs have been related to various diseases, such as different types of cancer, neurodegenerative diseases, autoimmune diseases, cardiovascular disease and other endocrine conditions. MiRNAs offer vast potential as biomarkers, contributing to early stage detection, disease classification, prognosis and prediction of treatment. Their involvement in various molecular pathways and gene expression implicates that early detection of specific miRNAs in biological fluids or tissues could correspond with the onset of certain diseases. Due to miRNAs’ relation to the coding genes whose expression they regulate, a large amount of information regarding the tissue origin of malignant cells could be generated. Moreover, these molecules also bear the ability to predict the course of a disease as well as its response to treatment. One major advantage miRNAs offer as biomarkers is that differences in their expression pattern are quantitative and can be measured with the aid of technology. Isolation of miRNAs is possible with most biological specimens, such as tissues, serum, plasma, blood, or urine. Another advantage they offer as biomarkers is that of durability and protection, due to microsomes and exosomes that contribute in developing a protective outer shell. Modern technological advances in nucleic acid amplification, sequencing, visualization and analysis offer the ability of miRNA identification in a variety of biological specimens. The available technological methods used for miRNA expression profiling are quantitative real-time polymerase chain reaction (qRT-PCR), use of microarrays, northern blotting, next generation sequencing (NGS) and in situ hybridization, with each preferred method following a predetermined extraction, visualization and quantification protocol for miRNA handling [30,31,32,33,34]. Future studies are needed, however, to further investigate the potential role of these molecules in disease detection.

### 2.6. MiRNA Implications in Diseases

Since their initial discovery, it became clear that miRNAs are expressed in a variety of cell types and that their expression patterns are tissue-specific; therefore, an assumption was made that they could be of great significance diagnostically and hold important prognostic value. Several miRNA studies have investigated miRNA expression patterns and biological pathways and have expanded current knowledge regarding miRNA implications in several diseases.

MiRNAs are, as already mentioned, a group of molecules that are widely involved in gene regulation and that therefore determine important cellular processes. A disease that relies mainly on uncontrollable cell growth is cancer, and the gene regulatory role of miRNAs was monitored as a possible incriminating factor by many studies. MiRNA-15 and miRNA-16 were the first molecules found to be downregulated in b-cell chronic lymphocytic leukemia patients. Moreover, expression profiling studies demonstrated that deregulated miRNA-21, miRNA-125, miRNA-145 and miRNA-155 increased the risk of breast cancer. MiRNAs seem to be involved in mechanisms surrounding metastasis and tumor invasion. For example, when downregulated, MiRNA-145 leads to primary gastric cancer and metastatic prostatic cancer progression. Studies also investigated the role of miRNA in tumorigenesis pathways. MiRNA-15 and miRNA-16 were found to repress anti-apoptotic gene b-cell lymphoma 2 (*bcl-2)* expression, while miRNA-155 plays a key role in the mismatch repair mechanism in colorectal cancer. These examples suggest that miRNA molecules involved in cancer pathogenesis will be capable of functioning as early stage disease markers as well as therapeutic targets in the future [31,32,35,36].

A field of knowledge that many studies have tried to expand is that of miRNA implications in viral diseases. When a host is infected, the viral agent also infects and modifies the host’s gene and miRNA expression patterns. MiRNAs have been found to be deregulated in Burkitt lymphoma infected by Epstein Barr Virus (EBV), and miRNAs were also identified in polyoma virus, adenovirus and several types of herpes viruses, such as HCV. Moreover, viruses have the ability to regulate miRNA expression, as maintenance of miRNA levels within the cell is crucial for viral infection and replication. Specifically, miRNA-28, miRNA-125, miRNA-150, miRNA-223 and miRNA-382 were found to be significantly downregulated in CD4+ T cells, resulting in high susceptibility to HIV. H1N1 influenza infection has also been linked to differentiated miRNA levels, as miRNA-21 and miRNA-223 have been measured and found to be elevated in lethal infections of H1N1 [37,38].

Cardiovascular diseases (CVDs) have been the number one cause of death worldwide for many years. Numerous studies have implicated the involvement of miRNAs in various cardiovascular diseases, as these molecules function as regulators of cardiac development and preservation. Among the CVD pathogenesis pathways in which miRNAs are involved are cardiac hypertrophy, myocardial ischemia, arrhythmias and atherosclerosis. MiR-1, miR-126, miR-197, miR-208 and miR-223 have been suggested by many studies as potential diagnostic biomarkers of acute myocardial infarction. Moreover, due to their pivotal role in CVD pathogenesis, miRNAs may function as a target for future treatments; specifically, miR-29, miR-92 and miR-328 have potential for use as therapeutic targets for acute myocardial infarction, ischemic disease and atrial fibrillation treatment [38,39].

Many immune-related diseases such as multiple sclerosis (MS), systemic lupus erythematosus (SLE), type 1/2 diabetes mellitus and non-alcoholic fatty liver disease (NAFLD) have been associated with miRNAs. MiRNA-145 was found to be upregulated, signaling a measure of distinction between MS patients and healthy controls; furthermore, miRNA-34, miRNA-155 and miRNA-326, retrieved from MS lesions, were also found to be upregulated. Low levels of miRNA-146 have been linked to increased risk for SLE, and diet-induced NAFLD has been associated with differential levels of specific miRNAs (miRNA-200, miRNA-429, miRNA-122, miRNA-451) [38,39].

Another category of diseases in which miRNAs seem to be involved is that of neurodegenerative diseases (ND). Their pathogenesis is complicated, and the exact mechanisms that affect the neuronal pathways still need to be elucidated. Therefore, many studies have tried to discover novel mechanisms and molecules involved in ND onset and progress. In that context, a miRNA profiling study with PD patients associated miRNA-26 and miRNA-30 levels in peripheral blood mononuclear cells with susceptibility to Parkinson’s disease (PD). Furthermore, miRNA-133 also has been linked to the pathogenesis of PD and, knowing that the miR-133-pituitary homeobox 3 (Pitx3) feedback loop plays a critical role in maintaining the dopaminergic neurons in the brain, the slightest deregulation in miRNA-133 levels might promote PD’s onset. Alzheimer’s disease (AD) is another ND in which miRNAs seem to play a pathogenetic role. Downregulation of miRNA-9 and miRNA-29 in AD patients has been strongly correlated with abnormally high levels of beta-site amyloid precursor protein cleaving enzyme 1 (BACE-1) protein, an important factor in AD’s pathogenesis [38,40].

Furthermore, various diseases have been also linked to altered miRNA expression patterns. Circulating miRNA-323 has been suggested as a subsidiary biomarker of human chorionic gonadotropin (hCG) and progesterone for the diagnosis of ectopic pregnancy. An abundance of miRNA molecules (miR-16, miR-23, miR-29, miR-107, miR-126, miR-191, miR-200, miR-362, miR-523) have been associated with Crohn’s disease, as their levels were found to be significantly elevated in blood samples collected from patients with Crohn’s disease compared to healthy controls. MiRNAs seem to be involved also in the progression of many kidney diseases. MiR-1, miR-133, miR-199 and miR-223 molecules measured in urinary samples of patients with autosomal dominant polycystic kidney disease were found to be dysregulated, therefore leading to an assumption that these specific miRNAs possibly play a part in the pathogenesis of this disease [35,38].

## 3. Role of miRNAs in T1DM

As already mentioned, miRNAs are non-coding RNA molecules that act as expression regulators in a variety of genes and are involved in other biological pathways, as well. One of these pathways is that of glucose metabolism. Balance in glucose excretion depends on pancreatic β-cell homeostasis to a fundamental degree. Differentiations in the expression patterns of genes specific for insulin secretion and resistance, in association with the three main T1DM pathogenetic mechanisms, have been studied widely and identified as the main points of miRNA involvement in the pathogenesis of T1DM.

Pancreatic islets consist of three cell types: α-cells, β-cells and δ-cells that produce glucagon, insulin and somatotastin. Through the production and release of glucagon and insulin, however, the pancreas regulates most of the metabolic pathways in the human body. Pancreatic β-cells, after elevation in blood glucose levels from feeding, induce insulin secretion, resulting in glucose uptake by peripheral tissues, and thereby reducing its blood levels. It is believed that a large amount of miRNA is involved in pancreas homeostasis; many studies have endeavored to identify the specific miRNA molecules that dysregulate pancreatic β-cell proper function, leading to their apoptosis [41,42,43,44,45]. One example is miRNA-21 (miR-21), a molecule that when overexpressed, was found to disrupt β-cell development in T1DM animal models [11]. MiR-21 regulates caspase levels; therefore, when overexpressed, it induces an increase in caspase-3 levels, eventually reducing cell count and viability. Moreover, studies reported that elevated levels of miR-21 result in increased β-cell apoptosis during diabetes development by specifically targeting the translation of the *bcl-2* gene [46,47]. Another miRNA molecule that is incriminated in β-cell dysfunction is miR-29, which, when increased in mouse and human pancreatic islets, was found to impair glucose-induced insulin secretion. Moreover, by targeting the antiapoptotic protein myeloid cell leukemia-1 (Mcl1), it promoted β-cell apoptosis and therefore pancreatic dysfunction in the early stages of T1DM development [48]. MiR-181 is found to be overexpressed in patients with T1DM when directly compared to healthy controls, and due to its negative correlation with C-peptide and SMAD7 levels, studies report that it potentially plays a significant role in pancreatic β-cell dysfunction [49,50,51]. MiR-7 and miR-124 are two molecules that were found to be implicated in the endocrine pancreatic differentiation pathway. Specifically, miR-7 was reported to inhibit the paire box 6 (*pax6*) gene, while miR-124 targets the transcription factor forkhead box A2 (Foxa2), and therefore, they are both negative regulators of α- and β-cell differentiation. An abundance of other miRNA molecules are involved in pancreas homeostasis disruption via gene targeting and protein cleavage [52,53,54]. Future studies to further investigate the mechanisms involved and pathways generated or blocked by miRNAs are deemed necessary.

Accumulating evidence has shown that miRNA molecules, due to their regulatory role in a variety of genes and involvement in many molecular pathways, are also involved in T1DM pathogenesis through more than one mechanism; immune system equilibrium is another factor that miRNAs may disrupt via association with multiple immunity-related genes. Regulation of immune cell differentiation, development and activation is determined in a major way by miRNA expression patterns. Islet-specific T cells and antibodies, when excited, act against β-cells, subsequently causing damage and T1DM onset. This attack on pancreatic islets includes CD4+, CD8+ T cells, macrophages, natural killer (NK) and dendritic cells (DCs), B lymphocytes and a plethora of chemokines and cytokines. Evidently, specific differentiations in the expression of individual miRNA genes possibly affect multiple immune responses. As T1DM is a progressive autoimmune disease, the protective and defensive role of B lymphocytes is significant. MiR-34a overexpression in diabetic mice has been linked with reduction in the capacity for B lymphopoiesis, through negative correlation with *Foxp1* gene expression—a gene essential for B lymphopoiesis, resulting in disturbance of pancreatic islet defense and sensitivity to damage [11,55]. Qualitative differential miRNA expression seems to directly affect the production of specific T lymphocytes as well. Overexpression of miR-23, miR-98 and miR-590 has been found to promote the production of auto-reactive CD8+ T cells that target islet antigens, through the inhibition of tumor necrosis factor-related apoptosis-inducing ligand (*TRAIL*), *FAS*, FAS ligand (*FASL*) gene expression. This indicates a gene silencing mechanism that promotes autoimmunity and subsequently T1DM onset. MiR-26 and miR-101 have been linked with differentiation of cells towards Th1 phenotype, via targeting histone 3 methyltranferase (Ezh2) and causing deficiency of set molecule. Furthermore, studies measuring miRNAs in peripheral blood mononuclear cells (PBMCs) have also reported association of miRNA expression patterns and autoimmunity. MiR-21 and miR-93 are involved in inflammatory and apoptotic signaling pathways and were found downregulated in PBMCs of T1DM patients, whereas miR-326, which targets significant modulators of the immune system—vitamin D receptor (VDR) and erythroblastosis virus E26 oncogenic homolog 1 (ETS-1)—was overexpressed in PBMCs of T1DM patients, further implicating the role of miRNAs in T1DM autoimmunity. MiR-15, miR-26 and miR-31 are also believed to alter T cell functions, as they are found overexpressed in pre-T1DM stages and have the capability of impairing T regulatory (Treg) cell survival [56,57].

Auto-antibodies against specific islet antigens IA-2, IA-2b and glutamic acid decarboxylase (GAD) are three of the major antibodies found in T1DM patients years before disease onset. MiRNAs may be involved in biosynthesis pathways of set auto-antibodies, as studies report that a cluster of 32 miRNAs modifies the expression sequence of these T1DM auto-antibodies [58]. Apart from interactions with auto-antibodies, miRNAs seem to be interrelated with several proinflammatory cytokines. Interleukin (IL) 1b and TNF-a were found to increase the expression of miR-21, miR-34 and miR-146, whereas miR-23 and miR-149, were downregulated when exposed to IL-1b and interferon (IFN) γ, consequently affecting β-cell apoptosis [48,56,57].

**Figure 2:**
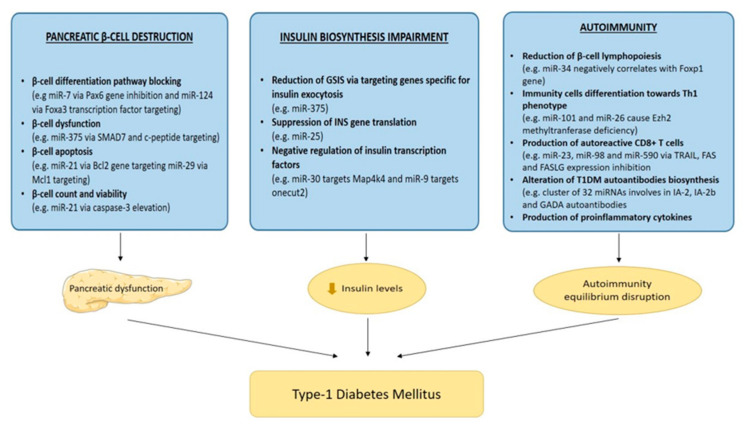
MiRNA-induced mechanisms in T1DM pathogenesis. MiRNAs via pancreatic β-cell destruction, insulin biosynthesis impairment and activation of the auto-immunity cascade leads to T1DM pathogenesis. Pancreas image from smart.servier.com (accessed on 15 September 2021).

T1DM onset can also be induced by impaired insulin biosynthesis and glucose-stimulated insulin secretion (GSIS). Recent studies reported that several miRNAs disrupt these pathways, negatively affecting insulin production and secretion. MiR-375 is a molecule that is found in abundance in pancreatic islets and acts by reducing glucose-induced insulin secretion by targeting Pdk1 kinase, as well as genes specific or relevant to insulin exocytosis, such as *Aifm1*, *Mtpu*, Gephyrin (*GPHN*) and *YWHAZ*. Moreover, miR-9 and miR-30 have also been related to insulin gene expression, either by negative regulation of Map4k4, a modulator of insulin transcription factor Mafa, or by directly affecting transcription factors of the insulin (*INS*) gene, such as onecut2, respectively. MiR-25 on the other hand, has been found to specifically target the *INS* gene, causing a suppression of its translation, reducing insulin secretion and further leading into T1DM pathogenesis [56,59,60]. A summary of the way in which miRNAs are involved in T1DM pathogenesis can be seen in Figure 2.

In addition, miRNA molecules have been associated with the pathogenesis of T1DM complications. Specifically, elevated miR-21 secretion in renal cells, induced by high glucose levels, causes renal cell hypertrophy and fibronectin expression in T1DM nephropathy. Renal fibrosis is also induced by another miRNA, miR-192, that when downregulated is associated with lower glomerular filtration rate (GFR) and tubulointerstitial fibrosis [11,61,62,63]. Furthermore, miR-126 is reported to be negatively correlated with vascular complications of T1DM, specifically retinopathy. MiR-144 expression patterns have been associated with diabetic heart disease, formation of reactive oxygen species and subsequently cell apoptosis, and miR-141 is found to play a significant role in the production of the inner mitochondrial membrane phosphate transporter in diabetic heart cells, directly affecting mitochondrial ATP production [64,65,66,67]. Although major progress has been accomplished, due to the knowledge gap surrounding miRNAs and their involvement in T1DM pathogenesis, further investigation of their implication in several T1DM stages, as well as in the regulation of key genes in disease onset and progression, is necessary.

## 4. Diagnostic Role in T1DM and Future Perspectives

MiRNAs, as already mentioned, are capable of reflecting the stage of ongoing disease, specifically T1DM, as they can be isolated from different cell types and biological fluids and demonstrate alterations occurring in disease progression, such as β-cell dysfunction and death. The goal of using miRNAs as T1DM biomarkers is that of early disease prediction, via miRNA profiling and quantification of its expression patterns, as well as discovering the triggering point of autoimmunity before autoantibody conversion. MiRNAs can be studied in several cell types such as serum, plasma, pancreatic tissue and urine, and due to their stability, they have already proved to be useful diagnostic tools. Many studies have targeted miRNA expression patterns in order to accumulate data regarding the stage of T1DM, the possible connection between up- or downregulation of existing miRNAs, and T1DM disrupted pathogenesis pathways. So far, numerous studies have measured the differential expression of miRNAs in T1DM patients and healthy controls and attempted to correlate these findings with functional alterations in specific stages of the disease. Elevated levels of miRNAs have been observed regardless of T1DM presence, opening the path for their use in disease prediction [68,69,70,71,72,73,74,75]. In non-diabetic children with the presence of autoantibodies, upregulation of miR-29, miR-21, miR-148, miR-339 and miR-425 was observed among others, with some of them correlating positively to certain autoantibody titers [70,76]. In other studies, patients with recent-onset T1DM appeared to express miR-27, miR-152 and miR-181 with significant consistency, while miR-375 levels were decreased [49,77,78,79]. Furthermore, correlation of these miRNAs to the abundance of different autoantibodies and to glycemic parameters enhances existing knowledge concerning miRNA association with residual β-cell function [80]. The long-term goal of miRNA application in the diagnosis of T1DM is the formation of a diagnostic panel of a miRNA group, potentially providing for diagnosis even at early asymptomatic stages of the disease. Taking into account findings that suggest that some miRNAs are significantly elevated while others are found to be significantly downregulated in pediatric T1DM patients, a score based on the diagnostic value of miRNAs could be developed and used as a basis that allows predictions of T1DM progression to be made. However, future studies are needed in order to further investigate the diagnostic role of miRNAs and provide more information regarding miRNA linkage to T1DM pathogenesis.

Since miRNAs were first discovered to be involved in T1DM pathogenesis, their gene regulation role and differential expression in body fluids and tissues rendered them capable of identifying individuals at risk of developing T1DM, as well as disease complications. However, on the basis of available data, no obvious advantages have been identified to replace the traditional biomarkers used to diagnose or predict T1DM. MiRNA measurement in circulation or in tissues can only be used so far as a complementary diagnostic method. Thus, regarding their efficacy in predicting the occurrence of T1DM and its complications, a systematic approach should be adopted with comparisons to existing biomarkers. Furthermore, with regard to their diagnostic value in T1DM, all current data and studies on potentially interesting miRNA biomarkers, in contrast to other biomarkers, need to be assembled and compared, and a consensus about the most appropriate miRNA molecules as T1DM predictors needs to be developed, so as to construct a pathway for their general use as diagnostic tools.

## 5. Bioinformatics Analysis

Candidate miRNAs and their hypothesized functions can be preclinically validated with the aid of bioinformatics analysis. It consists of a large and constantly growing body of databases that are able to predict miRNA targets. The aim of bioinformatics analysis is for independent algorithms to cooperate and predict the specific binding sites of miRNAs in their target genes. In this way, the extent of involvement by miRNAs in certain diseases, as well as the layout of the molecular pathways in which they interfere, can be pointed out and harnessed for development of novel biomarkers and therapeutic agents. The basic layout of bioinformatics analysis consists of several databases, such as miRTarbase, starBase, miRecords and TarBase. They involve miRNA-target interactions that were previously validated and in that way ensure that miRNAs bind strictly and specifically to a target [81,82]. After the desired targets have been selected, various independent algorithms are used in order to further predict the specific targets’ location in protein-coding genes, as well as the biological pathways that they are involved in. TargetScan is one of the first and best-known algorithms that has the capability of predicting gene targets of miRNAs, based on seed regions that are critical for binding [83]. In silico analysis also offers the advantage of calculating the power and strictness of miRNA–target binding, based on the measured free energy between them. Furthermore, another option in bioinformatics analysis is discovering the potential biological pathways that the selected miRNAs regulate, using the KEGG database, for example [84]. Therefore, in silico analysis offers the advantage of further complementing data that came from the experimental stage, by successfully predicting the binding sites of selected/studied miRNA molecules, the power of their bonds, as well as the biological pathways they regulate.

Since dysregulated miRNAs are in abundance in T1DM patients, the implications of miRNAs were further researched. Specifically, Assmann et al. performed a bioinformatics analysis to explore the genes targeted by dysregulated miRNAs, as well as the biological pathways that were impaired. Out of 45 dysregulated miRNAs in T1DM patients, a total of 9 miRNA molecules were differentially expressed in a group of recently diagnosed T1DM patients, when compared to healthy patients or patients with >5 years since diagnosis. Five of these miRNAs were then chosen for in silico validation, and the results indicated that miR-103a-3p was involved in the insulin signaling pathway, and miR-155-5p in immune-related responses of macrophages and activated T and B cells regulating corresponding genes. Apart from regulating cancer-related genes, bioinformatics analysis indicated that MiR-200a-3p is also involved in insulin secretion pathways. MiR-210-3p negatively regulated Foxp3 expression and targeted Tregs, while it was largely involved in various cancers and cardiovascular diseases. Last but not least, miR-146a-5p was involved in autoimmunity, with results from analysis indicating that when downregulated, it was associated with elevated anti-GAD antibodies concentration [85]. This study is an example of how bioinformatics analysis can be of great assistance to experimental research and specifically on miRNA implication in T1DM. It further elucidates the exact genes that miRNAs target, the expression patterns they regulate, and the surrounding biological pathways they impair, thus causing or consequently being involved in the pathogenesis of T1DM.

## 6. MiRNAs and Therapeutic Prospects

Understanding the role of miRNAs as gene regulators, as well as their involvement in various biological pathways, has led to novel applications for these molecules in diagnostics, with efforts being made to expand in therapeutics as well. Abnormal miRNA expression, as already mentioned, is associated with the pathogenesis of many diseases, and researchers have shifted their focus on developing clinical therapeutic strategies with miRNAs as the key elements. MiRNAs are either unregulated or downregulated, and that difference in their expression pattern is essential to the pathogenesis of certain diseases. The goal of treatment using miRNAs is to diminish their pathogenetic function. Inhibition of miRNA function in diseases is possible via anti-miRNAs, which are antisense oligonucleotides designed to bind to the desired miRNA and prevent interaction with its target [16]. In this context, antagomirs are a group of anti-miRNAs that are able to successfully block oncomirs in cancer. Antagomirs against miR-16, miR-122, miR-192 and miR-194 have already been tested in vivo in mammals with positive outcomes in multiple tissues [16,86]. Inhibition of miRNAs also has been achieved in vivo using locked nucleic acid (LNA), oligonucleotides or 2′-O-methoxyethyl phosphorothioate (MOE) modification, with reports of successful suppression of miR-122 in hepatitis C virus (HCV)-infected chimpanzees. MiR-122 is essential for HCV replication in liver and, with the use of an LNA-anti-miR-122 HCV viremia, was successfully blocked [16,87]. Another approach would be to block the pathogenesis pathway of miRNAs using tetracycline-inducible shRNAs that downregulate Drosha or Dicer proteins. This approach, however, is not without danger, as blockade of these molecules can affect the biogenesis of all miRNAs. Other miRNAs are downregulated in several diseases, and that loss of function also can be a therapeutic target. Restoration of miRNAs to their normal levels in the targeted tissue/cells can be accomplished with the use of synthetic RNA duplexes that resemble small interfering RNA (siRNA) molecules and therefore mimic miRNAs and their functions [84,88]. Although stability and delivery problems may arise, miRNA replacement with systemic administration of a miRNA mimic of miR-26 was used in mouse models with liver cancer, with highly positive results [16,89]. These therapeutic miRNA agents can be administered via either injection or intravenously. Delivery of these miRNAs can be accomplished via viral or non-viral systems. Retroviruses, adenoviruses and lentiviruses are able to transduce targeted cells with the desired miRNA gene, whereas non-viral systems include lipid-based systems, polymer-based systems and inorganic carriers [84,86,90,91,92].

Due to the positive outcomes with miRNA use in a variety of diseases including cancer, hepatitis C, and heart and kidney disease, an argument favoring applying miRNAs in T1DM therapy could be made. The two main approaches of miRNA therapy remain the same, with the first being restoring miRNAs that are downregulated in diabetes back to normal levels, using miRNA mimics, and the second being blocking the overexpression of specific pathogenetic miRNAs using miRNA inhibitors. Administration of miRNA mimics that regulate the induced reprogramming of pluripotent stem cells (iPSCs) can lead to regeneration of damaged insulin-producing cells and therefore restore insulin levels back to normal in diabetic patients [88,93,94,95]. Furthermore, blocking miR-21, miR-34 and miR-146 activity with the administration of anti-miRNA oligonucleotides might aid in preventing impairment of GSIS in specific cells [56,96]. Transfection of miR-23, miR-98 and miR-590 mimics into primary T-cells reduces FAS and TRAIL mRNA levels and therefore cytotoxic T cells that target pancreatic β-cells [16]. Another aspect in which miRNAs can be of assistance is that of drug resistance. Many reports have shown that miRNAs are associated with drug sensitivity and that by controlling their expression patterns, the sensitivity of a targeted drug can be shifted. So far, dysregulated miRNAs have been linked with drug resistance in breast cancer; however, their involvement can be expanded to other diseases as well. For example, insulin sensitivity in diabetes treatment could be associated with the expression pattern of specific miRNAs and therefore, hypothetically, synthetic miRNA agents can be used to manipulate drug resistance in diabetes treatment [84]. The knowledge that miRNAs are expressed in abundance in a variety of tissues and are significantly implicated in T1DM pathogenesis offers great promise for alternative treatments [97,98,99]. As dysregulation of miRNAs is observed in T1DM pathogenesis, restoring these miRNAs back to normal levels and using miRNA mimics or inhibitors could improve insulin production and secretion, as well as insulin sensitivity.

MiRNA use in therapeutics has enormous potential; however, many more studies and clinical trials are needed in order to confirm previous findings and shed light on unknown strands of miRNA involvement in treatment. Having knowledge of candidate drugs using miRNAs that are in clinical development or in phase 1 and phase 2 clinical trials for other diseases opens major new avenues of potential for the application of miRNAs in T1DM treatment. Although recent results have been encouraging, much more research and testing needs to be facilitated, in order to solidify and establish miRNA drugs in T1DM treatment.

## 7. Conclusions

MiRNAs are a recently discovered category of molecules that have since rapidly become a topic of research, with the number of miRNA-related studies growing exponentially. Their role as gene expression regulators has transformed them into emerging key factors in many physiological and pathological processes. Since miRNAs are found to be implicated in autoimmune pathways and insulin gene expression patterns leading to T1DM pathogenesis, all efforts have turned in the diagnostics direction, in order to provide a novel diagnostic biomarker capable of predicting T1DM in the early stages as well as its progression. While T1DM is a well-documented condition, it still offers a broad field for research, with future studies on the involvement of miRNAs in disease development and their value as diagnostic tools being a priority. It is anticipated that future years will expand our acquired knowledge of miRNAs and their applications in everyday clinical practice.

## Data Availability

Not applicable.
